# Two-step actions in infancy—the TWAIN model

**DOI:** 10.1007/s00221-019-05604-0

**Published:** 2019-07-19

**Authors:** Janna M. Gottwald, Gustaf Gredebäck, Marcus Lindskog

**Affiliations:** 1 Department of Psychology, Uppsala University, Box 1225, 75121 Uppsala, Sweden; 2 Department of Psychology, Durham University, South Road, Durham, DH1 3LE UK

**Keywords:** Movement duration, Reaching, Action development, Fitts’ law, Motor development, Model comparison

## Abstract

In this paper, we propose a novel model—the TWAIN model—to describe the durations of two-step actions in a reach-to-place task in human infants. Previous research demonstrates that infants and adults plan their actions across multiple steps. They adjust, for instance, the velocity of a reaching action depending on what they intend to do with the object once it is grasped. Despite these findings and irrespective of the larger context in which the action occurs, current models (e.g., Fitts’ law) target single, isolated actions, as, for example, pointing to a goal. In the current paper, we develop and empirically test a more ecologically valid model of two-step action planning. More specifically, 61 18-month olds took part in a reach-to-place task and their reaching and placing durations were measured with a motion-capture system. Our model explained the highest amount of variance in placing duration and outperformed six previously suggested models, when using model comparison. We show that including parameters of the first action step, here the duration of the reaching action, can improve the description of the second action step, here the duration of the placing action. This move towards more ecologically valid models of action planning contributes knowledge as well as a framework for assessing human machine interactions. The TWAIN model provides an updated way to quantify motor learning by the time these abilities develop, which might help to assess performance in typically developing human children.

## Introduction

Human object-directed actions are always part of a larger context and an action sequence; they seldom occur in isolation (Hesse and Deubel 2010; Marteniuk et al. 1987). When adults, for instance, reach for a cup, they do so to bring it to the mouth or to place it in a cupboard. Similarly, an infant might reach for a toy to manipulate it or to throw it somewhere. Here, we propose a novel model aimed at describing these real-world, two-step actions in 18-month olds. At this age, action capacities, the related kinematics, and the neural representations of the related limb dynamics are intensively developing (cf. Jansen-Osmann et al. 2002) and are not yet characterized by adult-like proficiency (Konczak and Dichgans 1997). We evaluate the model in the context of reach-to-place actions, where movement time is predicted by the precision demands (i.e., goal size and distance to the goal) of the involved action steps (i.e., reaching and placing). We show that the model, in addition to giving a good description of the data, can generate new insights into the nature of infant reach-to-place actions.

Developing a model for two-step actions in infancy was motivated by four issues: First, while established speed–accuracy models, such as the ones by Fitts (1954) or Welford et al. (1969), have been applied to describe infants’ actions (Gottwald et al. 2017; Zaal and Thelen 2005), they were originally developed to describe adults’ actions. During infancy, however, there is rapid development, both with respect to the actions that infants can perform and with respect to the accuracy of these actions (e.g., Chen et al. 2010). Using models primarily from the adult literature might, therefore, not be suitable to fully understand action development or to investigate the nature of two-step actions before they are fully mastered. Second, while existing models have been used to describe two-step actions (Gottwald et al. 2017), they were originally developed for single actions, such as finger pointing. For richer understanding of the development of the more ecologically valid two-step actions performed by infants, it is important to develop models that also consider the two-step nature of actions. Third, to date, there are few attempts at modeling infant behavior in general (but see, e.g., DeSantis et al. 2011; Munakata 1998), and specifically infants’ actions (Berthier 1996; Berthier and Keen 2006; Gottwald 2018; Gottwald et al. 2017; Zaal and Thelen 2005) despite the rich possibilities of such an approach. Finally, existing modelling studies with infants have comparably low sample sizes—around 10–12 infants per age group (e.g., Berthier and Keen 2006; Zaal and Thelen 2005). Studies with small sample sizes often have lower statistical power, reducing the likelihood of finding true effects (Button et al. 2013).

To date, there is little knowledge of the characteristics of infants’ two-step action production. By introducing this model, we, thus, address the fact that models describing infants’ actions are generally scarce, are for single actions, and are mostly evaluated on rather small samples. Before introducing our model, we first describe multiple (i.e., two or more) step actions and discuss related questions. We also briefly describe established models and their application in infancy research.

## Multiple-step actions

*Multiple*-*step actions* are actions consisting of two or more sub-actions—for example, reach-to-place actions, which consist of the two sub-actions (1) reaching and (2) placing (cf. Gottwald et al. 2017). Based on Bernstein’s conceptualization of action as goal-directed behavior with the goal as the center of action planning (Bernstein 1996; Grafton et al. 2008) and in contrast to non-goal-directed behavior, we can think of multiple-step actions as being to some extent planned before they are performed. The multiple steps of the action can be planned (or prospectively controlled) in two ways, below described by the example of reach-to-place actions: First, the steps could be planned and performed separately as single, isolated actions. That is, first the initial reaching action is planned. Once the reaching action has been initiated, the subsequent placing action is planned. Second, the steps could be planned as subordinated actions with one overall goal. In other words, both steps would be regarded as means to an overarching goal of the whole action sequence—for example, fitting an object into a box. Investigating which of these planning alternatives infants usually apply could help to understand the developmental mechanisms of multiple-step actions.

Previous studies have supported the second alternative that both action steps are planned with respect to the overall goal of the action sequence. The pioneering work by Marteniuk et al. (1987) demonstrated that adults perform their reach based on the parameters of the subsequent placing action: Adults reach faster for the same object when they are about to throw it in a wide container compared to when they are about to fit it into a small well. Similar results were shown for infants: 10-month olds perform their reach based on the parameters of the subsequent placing action (Claxton et al. 2003) and 14-month olds perform even the first part (i.e., the first movement unit; cf. von Hofsten 1979, 1991) of their reach based on the parameters of the subsequent placing action (Gottwald et al. 2017). These two infant studies, which measured reaching peak velocity and reaching duration, investigated two-step actions by varying the difficulty of the second action step while keeping the first step constant. The authors of both studies conclude that infants accommodate the parameters of the latter action when performing the former action; that is, they plan their first action step based on the parameters of the following action step. Thus, these studies (Claxton et al. 2003; Gottwald et al. 2017) suggest a prospective account of reach-to-place actions: the first action step (here: reaching) is influenced by what is happening next in the sequence (here: placing).

However, the results by Gottwald et al. (2017) also demonstrate that a version of Fitts’ law (Fitts 1954)—the Welford model (Welford et al. 1969)—describes the second action step well on its own as a single action. While both studies describe which information influences the first action step (i.e., reaching), neither study addresses what information influences the second action step (i.e., placing), when considered as a part of an action sequence (or two-step action). Thus, we do not have sufficient understanding of the planning and adjustment processes in multiple-step actions, or of the interplay of the relevant movement durations more generally. The next section will describe models of movement duration and their application in infancy research.

## Models of movement durations for actions

*Action difficulty* Generally, from reach onset at around 4 months, movement duration is a particularly important factor for describing infant reaching (Berthier and Keen 2006); whereas, movement duration is closely linked to the distance covered and task precision (Chen et al. 2010; Claxton et al. 2003; Gottwald 2018; Gottwald et al. 2017). Several previous models attempt to describe the movement durations of actions in the context of varying action difficulty; probably, the best known are Fitts’ law (1954) and its modification by Welford et al. (1969). Fitts’ law found, for instance, application in the pioneering work by Marteniuk et al. (1987). These authors demonstrated that adults’ reaching and pointing durations are influenced by the precision demands of the task, which we address as action difficulty: Adults’ reaches take less time when the target is larger than when it is smaller. It should be noted, however, that the models by Fitts (1954) and by Welford et al. (1969) are not the only models suggested in the literature. In fact, a review by Plamondon and Alimi (1997) outlines more than ten models describing the movement durations of various types of actions. We now outline the gist of Fitts’ law and the Welford model. However, for parsimony, when evaluating our model, we include four additional models (see Table 2) that are applicable to our reach-to-place task. Common to all models is that they predict the time it takes to move to a target (movement time, MT) as a function of the distance to the goal (*A*) from a given starting point and the size of the goal (*W*).

*Fitts’ Law* Fitts’ law describes the relationship between action difficulty and movement time. It states that the movement time (MT) required to reach a goal area is a function of the distance to the goal (*A*) and the size of the goal (*W*) given by MT = *a* + *b* × log_2_ (2*A/W*), where log_2_ (2*A/W*) is the spatial relative error or the index of difficulty and *a* and *b* are empirical constants. In other words, the easier an action becomes, the less time is required to successfully perform it.

*Welford’s model* Several modifications of Fitts’ law have been formulated (for a review see Plamondon and Alimi 1997). In contrast to other models (see Table 2), the version by Welford et al. (1969) allows for the evaluation of separate contributions of goal size and goal distance to movement time: Here, movement time is given by MT = *a* + *b*_A_ × log2 (*A*) + *b*_S_ × log_2_ (1/*W*). The most crucial difference to Fitts’ law is that this model treats goal distance and goal size as independent factors giving independent contributions to movement time.

*Application in infancy research* Two previous studies explicitly tested whether infants’ single actions (reaching) are well described by Fitts’ law. Zaal and Thelen (2005) demonstrated that 7- to 11-month-old infants reach more slowly to smaller objects than larger objects. By regressing movement time on goal size, the authors predicted 45% of the variation in movement times. Gottwald et al. (2017) applied Welford’s model to 14-month-old infants’ two-step actions (reach-to-place sequences) and demonstrated that placing actions were well described by this model. Similar to the results of Zaal and Thelen (2005), Gottwald and colleagues showed that goal distance and goal size predicted 48% of the variation in movement time. In this study, Welford’s model was chosen over Fitts’ to evaluate the separate contribution of distance and size on movement time. Both parameters contributed significantly. However, when action difficulty was held constant, as it was for the first action step (reaching), Welford’s model explained only 6% of the variation in movement time. Note that the amount of explained variance in reaching time in the above-mentioned studies (45% and 48%) is relatively high given the generally high variability in infant behavior and particularly infant motor behavior (Fagard and Lockman 2005; Thelen et al. 1993, 1996).

## TWAIN—a model for two-step actions in infancy

The results of Zaal and Thelen (2005) and Gottwald et al. (2017) are important because they show that it is possible to model the movement durations of infants’ actions. However, as noted above, both studies considered only the special case of single-step actions (Zaal and Thelen 2005) or limited the analysis of two-step actions to single-step components (Gottwald et al. 2017). We, therefore, propose a new model aimed at describing movement durations in the two-step actions of a reach-to-place task performed by infants. For clarification, by reaching duration, we mean the duration of the initial reach for the object (action step 1) and by placing duration, we mean the duration of the whole placement phase (action step 2).

Our model takes three observations as its starting point. First, previous studies indicate that placing movements can be reasonably well described by considering goal distance and goal size as independent factors contributing to movement time, as in Welford’s model. Second, although used to model parts of action sequences (as e.g. in Gottwald et al. 2017), all the previous models describe single-step actions. They do so even though the reach-to-place task, like most real-life actions, includes more than one step. Third, it is reasonable to assume that the duration of previous actions might also influence the duration of subsequent actions. Accordingly, we suggest that the duration of the placing movement in a reach-to-place task is influenced by (1) the distance to the goal and the size of the goal, as in Welford’s model, and (2) the duration of the reaching movement. This notion is formalized in the following model for two-step actions in infancy:

$$ {\text{MT}}_{\text{p}} = a + b_{\text{A}} \log_{2} A + b_{\text{W}} \log_{2} \frac{1}{W} + b_{\text{MTr}} \log_{2} {\text{MT}}_{\text{r}} , $$

where ${\text{MT}}_{\text{r}}$ is the movement duration for the reach preceding the placing movement, *a* and *b* are empirical constants, *A* is goal distance, and *W* is goal size. Below, we describe an experiment designed to evaluate the applicability of our model while also evaluating its performance against a set of previously proposed models.

## Materials and method

### Participants

The final sample included 61 18-month olds (age range = 529–561 days, *M* = 542 days, SD = 8, 34 boys, 26 girls). A further nine infants were tested but excluded from analysis due to unwillingness to perform the task (*n* = 3), lack of compliance with inclusion criteria (i.e., they performed no valid trial, *n* = 2) or technical error (*n* = 4). Participants were recruited from the lab’s list of parents who expressed interest in participating in research studies with their child. Informed consent was obtained from both parents of all participants. For participation, parents received a gift voucher of 100 Swedish Crowns (≈ 10 Euro).  All procedures involving human participants were performed in accordance with the ethical standards of the regional ethics committee and the 1964 Declaration of Helsinki (World Medical Association 2013) and its later amendments or comparable ethical standards.

### Materials and procedure

**Fig. 1:**
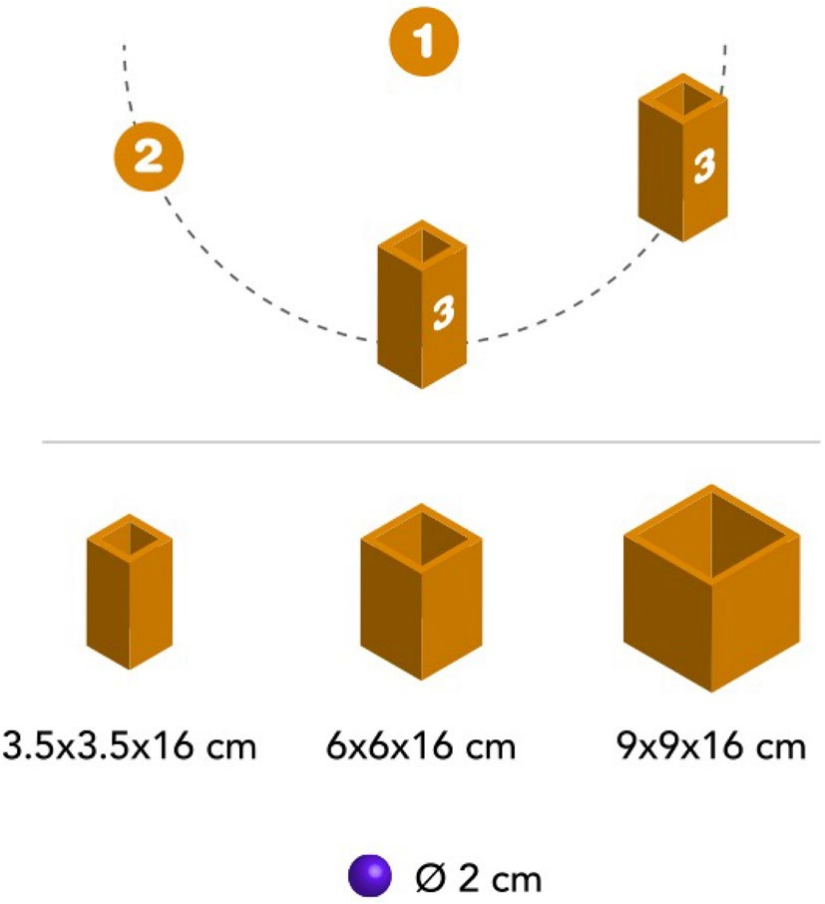
Material and procedure. The children placed their right hand on the staring area (1), reached across 20.5 cm for the object (2), and placed it into a box (3), which was either located in a short (12 cm) or long distance (37 cm) to the object’s pick-up area. Every child performed all possible size–distance combinations

The task adapted from Claxton et al. (2003) and Rosander and von Hofsten (2011) has previously been used to study prospective motor control in 14-month olds (Gottwald et al. 2017) and was part of a procedure of four tasks, as reported by Gottwald et al. (2016). Including breaks and instructions, the procedure took approximately 15 min (see Fig. 1 for illustration and Gottwald et al. (2016) for further details).

The caregiver sat at a table with the infant on their lap, facing the experimenter. The task was to reach for a toy (2 cm in diameter) and place it in a box. Whereas the difficulty parameters of the reaching step were kept constant, the parameters of the subsequent placing step were varied by goal size and goal distance. We used three differently sized boxes that were 16 cm in height with inner dimensions of either 9 by 9 cm (large), 6 by 6 cm (medium), or 3.5 by 3.5 cm (small). First, the experimenter demonstrated with the toy and one of the boxes. Thereafter, she placed the object and box at defined positions on the table. The object and boxes were arranged in a half-circle around the child, that is within their reaching space (von Hofsten 1982). The caregiver then reached for the toy and placed it into the box. This demonstration was done twice. In the following test trials, the experimenter again presented the child with the object and one of the boxes and placed them on the table. The distance between the object and box was either 12 cm or 37 cm. Children were instructed to place their right hand on the starting area (5 cm in diameter) and to place the object in the box. 18 trials were performed in a counterbalanced order in blocks of three identical trials and continued until the child lost interest in the task.

### Data recording

Data were recorded with an eight-camera passive motion-capture system (Qualisys Motion Capture Systems, Gothenburg, Sweden) that tracked the motion of markers (0.6 cm in diameter) attached to the infants’ hands at a sampling rate of 240 Hz. Every session was filmed by a video camera.

### Data analysis

Videos were coded for the beginning and end of the two action steps (reaching and placing) using Qualisys Track Manager (Qualisys, Gothenburg, Sweden). We visually identified the last frame before the start of the movement of the right hand, the first contact between hand and object, and the last frame before letting the object go to place it in the box. A second-rater double-coded 29% of the videos; inter-rater reliability (ICC) was 0.97. Data of the right hand were analyzed (left reaches occurred rarely). Valid trials were considered direct reaching movements without interference from the caregiver and meant moving from the marked area to the object followed by direct placing movements. Only successfully completed reach-to-place sequences were considered as valid. The movement durations of all valid reach-to-place were extracted using TimeStudio, a plug-in-based toolbox for MATLAB (Nyström et al. 2016). The data and analysis are available publicly within the TimeStudio Project (timestudioproject.com, code: uwid ts-6b9-ado). The data can be additionally found within the Open Science Framework (10.17605/osf.io/5xz6m).

## Results

We standardized movement time for place duration and reach duration separately and removed any movement times with |*z*| > 2.5 on either place or reach duration. This procedure removed 3.03% of data points.

### Placing movement duration

On average, the duration of the placing action (movement time) was 1.32 s (SD = 0.55; see Table 1).

**Table 1:** Mean reach and placement duration in seconds (SD) in the six combinations of distance (cm) and goal size (cm) arranged from the easiest (first row, short distance–large goal) to the most difficult action (last row, long distance–small goal)

Distance (*A*)	Size (*W*)	Reach duration	Placement duration
12	9.0	0.840 (0.192)	1.08 (0.59)
12	6.0	0.802 (0.161)	1.08 (0.38)
12	3.5	0.846 (0.202)	1.48 (0.51)
37	9.0	0.845 (0.198)	1.23 (0.47)
37	6.0	0.848 (0.172)	1.37 (0.55)
37	3.5	0.849 (0.195)	1.64 (0.59)

### Modelling placing movement durations

We evaluated the viability of the TWAIN model for describing placing movement durations in two steps. First, we fitted the model to the placing movement duration data and evaluated its performance. We argued that for the model to be a good candidate to describe a two-step action, it should explain a significant amount of variance and all three predictors (goal size, distance to goal and movement duration) should be significant. Second, we evaluated if the TWAIN model gave the best account of the data by comparing this model to six previously suggested models. All model fitting was done in R (R Core Team 2017). We used the R stats package to fit each of the models separately to the placing movement duration using nonlinear least squares curve fitting with the nls function. All models were fitted on individual level data; that is, on the individual mean placement and reach duration in each of the six distance and size combinations.

**Fig. 2:**
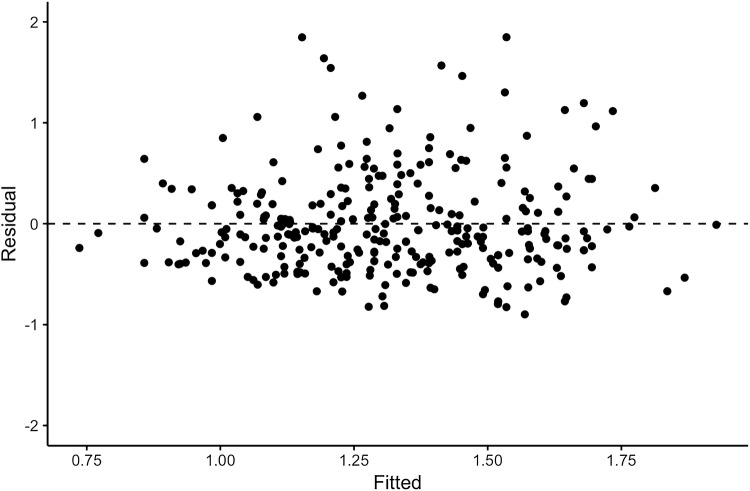
Residual plot for the fitted TWAIN model

*Performance of the TWAIN model* Fitting the TWAIN model to the placing data revealed a significant model *F*(3, 296) = 19.53, *p* < 0.001 with three significant predictors (*b*_A_ = 0.12, *p* = 0.002, *b*_W_ = 0.29, *p* < 0.001, *b*_MTr_ = 0.35, *p* < 0.001). The model explained approximately 17% (*R*^2^ = 0.165) of the variance in the placing movement duration. Although this is smaller than the 45% to 48% observed in previous studies (Gottwald et al. 2017; Zaal and Thelen 2005), it was more than Welford’s model explained in the current data set (12.5%). This indicates that the TWAIN model can give a good description of the data. Figure 2 shows a residual plot for the TWAIN model. As can be seen in the figure, the residuals are close to symmetrically distributed around zero with no indication of outliers, a non-linear relation, or heteroscedasticity, which the model could not capture. This further indicates that the model gives a good description of the data.

**Fig. 3:**
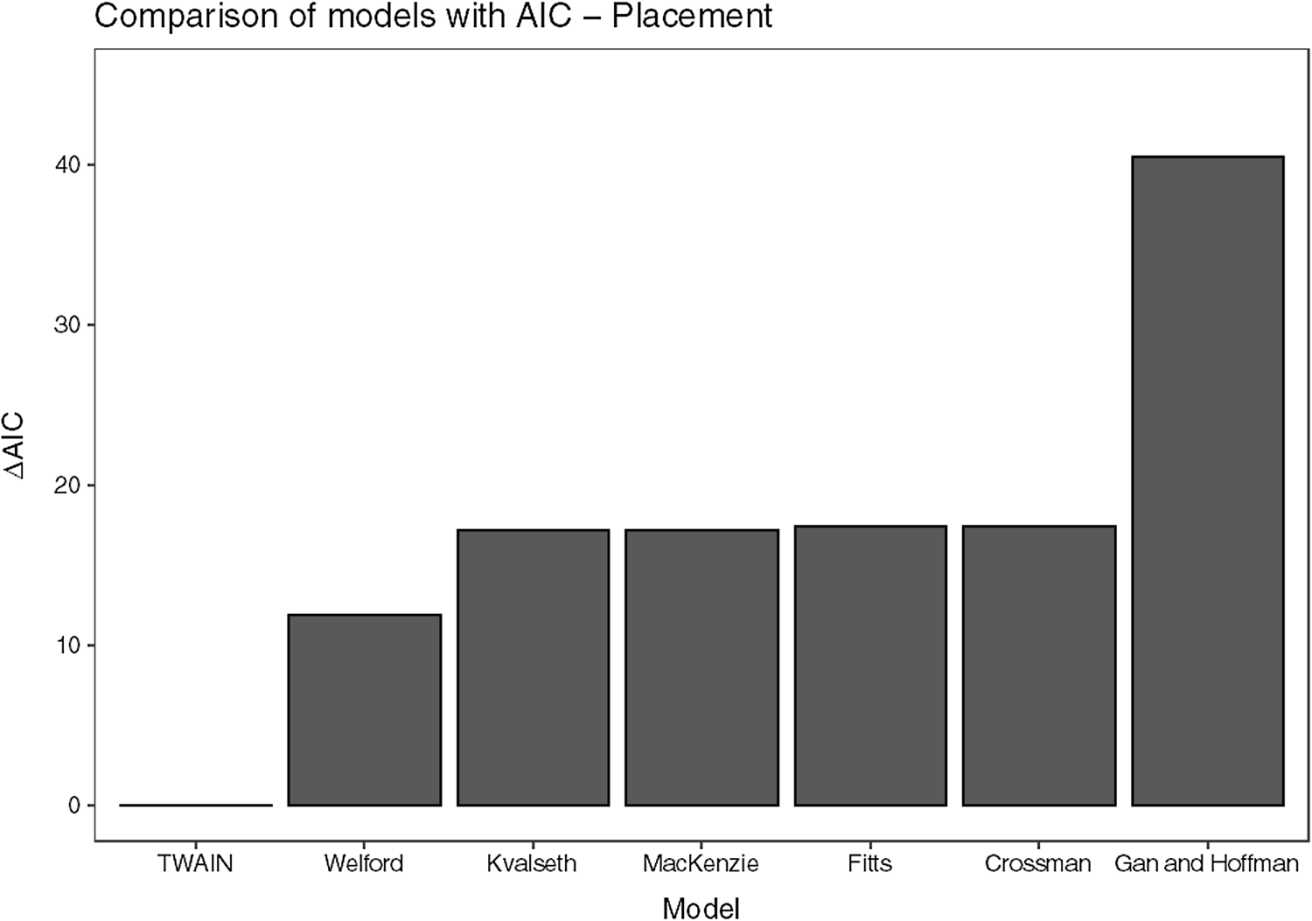
AIC relative to the best fitting model (ΔAIC = 0) for the seven models

*Model comparison* To compare the TWAIN model with six other previously proposed models, we calculated each model’s Akaike Information Criterion (AIC). The AIC is a measure of model fit that penalizes models with more parameters, thus making it possible to compare models of different complexity. A lower AIC indicates a better fit of the model to the data. We used nonlinear curve fitting to ease the comparison between the models and because one of the models (Kvålseth 1980) gives *MT* as a nonlinear function of *A* and *W*. Table 2 summarizes the model fits in terms of AIC for each of the six models. The results are also illustrated in Fig. 3, which shows the difference in AIC ($\Delta$ AIC) to the best fitting model for the second action step—the placing movement duration. This analysis shows that the TWAIN model gives the best account of the placing duration data (AIC = 453), even when considering that it has an additional free parameter to the next best fitting model, which was Welford’s model (AIC = 465).

**Table 2:** Model fit in terms of AIC and *R*^2^ for the placing data for the models of movement durations

Source	Model	AIC	*R* ^2^	ΔAIC
TWAIN (current model)	$$ \begin{aligned} MT_{{\text{p}}} & = a + b_{{\text{A}}} \log _{2} A \\ & \quad + b_{{\text{W}}} \log _{2} \frac{1}{W} + b_{{\text{R}}} \log _{2} {\text{MT}}_{{\text{r}}} \\ \end{aligned} $$	452.59	0.17	0.00
Welford et al. (1969)	${\text{MT}} = a + b_{\text{A}} \log_{2} A + b_{\text{W}} \log_{2} \frac{1}{W}$	464.53	0.13	11.94
Kvålseth (1980)	${\text{MT}} = a\left( {\frac{A}{W}} \right)^{b}$	469.77	0.10	17.18
MacKenzie (1989, 1992)	${\text{MT}} = a + b\log_{2} \left( {\frac{A}{W} + 1} \right)$	469.83	0.10	17.24
Fitts (1954)	${\text{MT}} = a + b\log_{2} \frac{2A}{W}$	470.09	0.10	17.50
Crossman (1956)	${\text{MT}} = a + b\log_{2} \frac{A}{W}$	470.09	0.10	17.50
Gan and Hoffman (1988)	${\text{MT}} = a + b\sqrt A$	493.10	0.03	40.51

## Discussion

From an early age, infants carry out multiple-step actions in different contexts. Previous studies have used models in an effort to describe such actions (for review see Gottwald et al. 2017). In this paper, we proposed a novel model—the TWAIN model—to describe the movement durations of two-step actions in a reach-to-place task in infancy. The proposal of this model was motivated by previous modeling attempts (Gottwald et al. 2017; Zaal and Thelen 2005), which used models originally developed for single actions and validated on adults (e.g., Fitts’ law). The TWAIN model was specified as to integrate previous findings suggesting that placing movements can be described by considering goal distance and goal size as independent factors, both contributing to movement time (Gottwald et al. 2017). The TWAIN model also assumes that the duration of previous actions can influence the duration of subsequent actions. Similar to previous studies, we evaluated the TWAIN model in the context of a reach-to-place task. This was done by first examining the model’s ability to explain the data in the reach-to-place task and second by comparing its performance to that of existing models. Our TWAIN findings are: First, fitting the TWAIN model to the placing duration data indicated a significant model with three significant predictors that explained approximately 17% of the variance in placing duration. This suggests that the TWAIN model can give a reasonably good explanation of the durations of the reach-to-place task in the current experiment. Second, for these data, the TWAIN model outperformed six other previous suggested models, two of which (Fitts’ law and Welford’s model) have previously been used to model infant data. Thus, not only did the TWAIN model give a good description of the data, it also gave the best description when compared to other models.

Beyond looking at actions in isolation, the TWAIN model aimed to address actions in a more ecologically valid fashion by considering that most actions are context dependent, that is part of a sequence of action steps. We show that including parameters of the first action step, here its duration, can greatly improve the description of the second action step.

Further, our attempt at describing two-step actions with the TWAIN model enables new insights into the characteristics of infant reach-to-place actions. Previous studies proposed a prospective account of two-step actions (Claxton et al. 2003; Gottwald et al. 2017, for review see Gottwald et al. 2017): The first action step is influenced by the second step; indicating infants’ motor planning in action sequences. The current study adds to these findings by demonstrating that the first and the second action steps are more complexly related: the second action step is also influenced by the first step. In other words, the slower the infants reached for the object, the slower they subsequently placed it. Thus, in addition to the prospective effects, there are carry-over effects (cf. Hansen et al. 2015) or transfer effects (cf. Jansen-Osmann et al. 2002) when infants carry out multiple-step actions.

In the context of our finding, there are two possible explanations of these carry-over effects. First, infants may use proprioceptive and visual feedback from their reaching movements to control their subsequent placing movements. That is, longer reaching durations lead to longer placing durations, because infants tailor their future actions based on their current action. Second, the case could be much simpler: maybe the speed at the beginning of the action sequence lays the foundation for the whole action sequence. That is, longer reaching durations lead to longer subsequent placing durations because infants engage in one action flow: the movement speed of the first action step transfers to the second action step. Hence, the infants could maintain the speed chosen for the first action step for the subsequent action step, without further adaptations. In this context, there could be individual differences in general reaching speed or state-based influences at work. This does, however, not undermine the validity of the model or its implication for how to interpret two-step actions in infancy.

The interdependence of feed-forward control and feedback control of movements in multiple-step actions and its change over time is an interesting question. Future research should address this further by disentangling both possible explanations for our results. Jansen-Osmann et al. (2002) demonstrated that reaching in middle childhood involves the use of forward and inverse models, which might also be the case in younger children (as here for 18-month olds). The current study does not investigate speed/accuracy demands in the traditional way (as in studies with older children and adults) by instructing participants to perform the two-step action as quickly and accurately as possible. This is not possible with such young participants. We did encourage the infants to move quickly, but we did not systematically manipulate speed demands. To do this with infants, one could introduce speed demands (beside the here applied accuracy demands) using objects moving at different speeds instead of static objects (as, e.g., Ekberg et al. 2016).

Five caveats should be mentioned. First, a dropout rate of 13% for the sample is—given the manifold challenges posed by behavioral studies with 18-month olds—relatively low. However, questions might arise if we effectively excluded children who were not capable of movement planning in two-step actions, and thus have less generalizable results. However, since only 3% (*n* = 2) of the children were excluded due to the criterion of having performed no valid trial, this possible limitation can be laid aside. Second, since the mentioned valid trials contain successful two-step actions, we cannot draw conclusions about unsuccessful movements. Thus, the kinematics underlying unsuccessful two-step actions are unknown. Future research should address the question: how unsuccessful action sequences can be accurately described, and if there are regularities to the interdependence of the durations of the relevant action steps. Third, besides carry-over effects within one action sequence, there could be carry-over (or transfer effects) between trials. The placing action of the prior trial could influence the reaching action of the subsequent trial. We partly addressed this by counterbalancing the trials in blocks. However, since almost none of the participants contributed data from all trials to the data set, this study cannot answer the question of carry-over effects between trials. Fourth, when fitted to the data, the TWAIN model could explain approximately 17% of the variation in placing duration. Given the high intra-individual variability of infant data (Gampe et al. 2015; Thelen 1995), this is a reasonably high amount of variance explained. However, it is still lower than in previous modeling attempts (Gottwald et al. 2017; Zaal and Thelen 2005). One possible reason for this discrepancy is that the current study (*n* = 61) used a within-subjects design where infants performed the same task in six different condition. This provided more data points than in the previous studies. For comparison, Gottwald et al. (2017; *n* = 37) used two within-subject conditions (goal size) and two between-subject conditions (goal distance). Zaal and Thelen (2005; *n* = 30) used two between-subject conditions (goal size) and let infants reach from whatever distance they wanted. Although the design of the current study allows for a more fine-grained analysis of the influence of goal size and distance, it might also introduce more noise to the measurements. Put simply, it is easier to fit a linear model to two than to six conditions. A further implication of this is that both Fitts’ law and the Welford model explained considerably less variance in the current data set than in previous studies. It should be noted, however, that the TWAIN model provided a better fit to the data than both Fitts’ law and the Welford model. Future studies should evaluate the performance of these models under conditions allowing for a fine-grained analysis and minimal measurement noise. Finally, the current study included only one age group. Future studies should apply the TWAIN model to movement durations in two-step actions of different (older) age groups to see whether the model improves or whether a different model applies.

The here-proposed TWAIN model could find application in designing human–machine interfaces and software interfaces, where multiple pointing, shifting and pressing movements are usually involved. The model might, therefore, contribute to technical development. Further, providing a good description of multiple-step actions in typically developing young children, the TWAIN model could be used to compare and evaluate performance of multiple-step action durations in typically and atypically developing children.
